# The Prevalence of the EML4-ALK Fusion Gene in Cytology Specimens from Patients with Lung Adenocarcinoma

**DOI:** 10.1155/2020/3578748

**Published:** 2020-12-23

**Authors:** Didik S. Heriyanto, Ika Trisnawati, Evan G. Kumara, Vincent Laiman, Fara S. Yuliani, Auliya S. B. Sumpono, Rita Cempaka, Marcellus, Eko Budiono

**Affiliations:** ^1^Department of Anatomical Pathology, Faculty of Medicine, Public Health, and Nursing, Universitas Gadjah Mada–Dr. Sardjito Hospital, 55281 Yogyakarta, Indonesia; ^2^Department of Internal Medicine, Faculty of Medicine, Public Health, and Nursing, Universitas Gadjah Mada–Dr. Sardjito Hospital, 55281 Yogyakarta, Indonesia; ^3^Department of Pharmacology and Therapy, Faculty of Medicine, Public Health, and Nursing, Universitas Gadjah Mada, 55281 Yogyakarta, Indonesia

## Abstract

**Background:**

Under the National Comprehensive Cancer Network (NCCN) guidelines for non-small-cell lung carcinoma (NSCLC), anaplastic lymphoma kinase (ALK) gene rearrangement is required to be assessed. However, data showing the prevalence of the ALK rearrangement is still deficient and is not yet available in Indonesia. This study used direct smear preparation from transthoracic needle specimens that are minimally invasive. The main objective of the study is to identify the prevalence of the ALK fusion rearrangement gene in cytological specimens.

**Materials and Methods:**

A total of 35 direct smear preparations diagnosed as lung adenocarcinoma and EGFR mutation negative were involved in this study. The samples were taken between 2017 and 2019. These samples were examined for EML4-ALK fusion rearrangement gene using qRT-PCR. The EML4-ALK rearrangement status was determined by qRT-PCR with high-resolution melting (HRM) analysis.

**Results:**

A total of 28 (80%) samples were from males, and 7 samples were from females. Seven (20% 95% CI: 8.4%-36.9%) samples were EML4-ALK rearrangement positive. The average age of the patients was 63.5 years old. The most common sites of metastasis in this study were pleural cavity, bone, liver, and CNS.

**Conclusions:**

qRT-PCR successfully identified EML4-ALK fusion rearrangement in direct smear preparations of lung adenocarcinoma. Direct smear samples can be used for EML4-ALK rearrangement detection using qRT-PCR. The EML4-ALK rearrangement gene has high prevalence in selected lung adenocarcinoma and EGFR mutation-negative populations. ALK inhibitors in lung cancer can be openly considered for use in Indonesian patients to improve the outcome of this subset of patients.

## 1. Introduction

Lung cancer has the highest rate of morbidity and mortality of all cancer types. Each year, lung cancer causes more deaths than colon cancer, breast cancer, and prostate cancer combined. Lung cancer is one of Indonesia's leading causes of deaths. Ministry of Health data shows that 6% of the population in Indonesia has had a cancer diagnosis [1, 2].

One of the classifications of lung cancer is non-small-cell lung carcinoma (NSCLC). The adenocarcinoma subtype of NSCLC is mainly found in Asian women. Lung adenocarcinoma harbours numerous molecular abnormalities, including mutation of the epidermal growth factor (EGF), rearrangement of the gene for anaplastic lymphoma kinase (ALK), and mutations for Kirsten rat sarcoma viral oncogene homologue (KRAS). Targeted treatment with these molecular markers has revolutionized precision medicine. In recent years, lung cancer driver genes have been increasingly identified and confirmed with the rapid development of molecular biology, encouraging the advent of molecular targeted drugs and ushering in the era of targeted drug therapy [1, 3].

All laboratories that examine lung cancer specimens are advised to test for mutations in a minimum of one set of genes, namely, epidermal growth factor receptor (EGFR), ALK, and ROS1. As appropriate specimens for lung cancer biomarkers, pathologists may use either cell blocks or other cytological specimens [4]. ALK gene rearrangement has been identified as a particular molecular subtype in NSCLC. Patients with this rearrangement responded to ALK tyrosine kinase inhibitors (TKI) [5]. The echinoderm microtubule-associated protein-like 4- (EML4-) ALK fusion oncogene is one of the latest molecular targets in NSCLC. EML4-ALK shows high in vitro and in vivo oncogenic activity [6]. A recent study shows that alectinib is a significant therapeutic choice for patients with advanced ALK-positive NSCLC. Given its effectiveness and tolerability, the National Comprehensive Cancer Network (NCCN) recommends alectinib as a preferred first-line therapy choice [7].

The diagnosis of lung cancer is frequently made using minimally invasive procedures. Various sampling techniques are available to procure cytologic samples for evaluation in lung malignancies. The use of cytological specimens for molecular testing can be done when a diagnosis of pulmonary carcinoma is established [1]. In Indonesia, the majority of lung cancer cases are found in late stages, and cytological specimens are common sources for diagnostic practices, especially in tertiary hospitals [8]. The average success rate for the assays that use cytological specimens is very high. This is because cytological samples are almost always fixed and processed immediately after sample collection, which is usually performed without delay. Thus, cytological samples should have well-preserved content, and hence, they offer the possibility of testing higher-quality nucleic acids for reliable molecular outcomes [9].

Different types of cytology samples have been demonstrated to be appropriate for molecular research, including fine-needle aspiration (FNA), bronchoscopy-guided FNA, direct aspirations, and pleural effusion [10]. In a limited number of cytology specimen condition, the use of these approaches may be preferable [11]. Cytological specimens and small histopathological specimens appear to perform similarly, with high feasibility of molecular testing demonstrated irrespective of the diagnostic modality. Perhaps unexpectedly, FNA has been shown to be comparable to core biopsy for molecular testing following radiologic transthoracic needle biopsy [12].

Previous studies have demonstrated good concordance between cytology-based and histology-based testing for both EGFR and ALK [5, 10]. DNA of good quality can be collected from stained smears, fresh fluids, and cell blocks. Variations in the fixating technique favour alcohol fixatives rather than formalin for mutational studies. Cells from cytological smears can be extracted from the glass slides by scraping for DNA extraction. Scraping has been shown to produce substantially more DNA than cell lifting [10].

This study uses cytology specimens, which have previously been shown to be reliable in mutational studies and are minimally invasive. This study is expected to facilitate the development of optimal diagnostic and treatment strategies to improve the prognosis of this subset of patients. The present study is aimed at examining the prevalence of the EML4-ALK rearrangement gene in pulmonary adenocarcinoma in direct smear specimens.

## 2. Materials and Methods

This was an observational study with a cross-sectional design using consecutive sampling. This study initially collected 274 cytology samples diagnosed with primary NSCLC between sampling period of 2017 and 2019. These were direct smear cytology specimens obtained by transthoracic needle aspiration at the Department of Anatomical Pathology, Faculty of Medicine, Public Health, and Nursing at the Universitas Gadjah Mada, Yogyakarta, Indonesia. Of those samples, only 162 samples diagnosed with adenocarcinoma subtype of NSCLC were selected. Of those lung adenocarcinoma samples, only those with EGFR mutation-negative samples were involved in this study. In addition, the number of remaining available tumour cells to be included in this study after the EGFR test was at least 500 cells. Metastatic lung cancer from other organs and samples showing predominant necrosis were excluded. All Diff-Quick^TM^ stained slides were examined, and the specimens with well-preserved tumour cells were selected. In the end, this study included 35 cytological direct smear specimens fulfilling the criteria. The samples were then examined for the EML4-ALK rearrangement at the Department of Anatomical Pathology, Faculty of Medicine, Public Health, and Nursing, Universitas Gadjah Mada Yogyakarta, Indonesia. All specimens were collected with the approval of the ethical committee at the Medical and Health Research Ethics Committee (MHREC), Faculty of Medicine, Public Health, and Nursing, Universitas Gadjah Mada–Dr. Sardjito General Hospital, Yogyakarta, Indonesia (Ref. No.: KE/FK/0532/EC/2020).

### 2.1. RNA Extraction

RNA extraction was performed on scraped smear preparations using the Ribospin^TM^ II RNA Purification Kit (Cat. No. 314-150) according to the manufacturer's instructions.

### 2.2. Real-Time Polymerase Chain Reaction (qRT-PCR)

The RNA samples were assayed using the AmoyDx® EML4-ALK Fusion Gene Detection Kit (Amoy Diagnostics, Xiamen, China) according to the manufacturer's instructions. Quantitative PCR was performed using Bioneer Exicycler^TM^96 Real-Time Quantitative Thermal Block. The PCR condition was as recommended by the manufacturer. The EML4-ALK rearrangement status was determined by qRT-PCR with high-resolution melting (HRM) analysis.

### 2.3. Data Analysis Plan

All statistical analyses were performed using Microsoft Excel 2020 for Mac OSX. Categorical data were expressed as frequency and percentage, and quantitative data were expressed as mean. Univariate analysis is performed to obtain an overview of each variable. Confidence interval for binominal variable was calculated with Clopper-Pearson 95% CI.

## 3. Results

The characteristics of the samples are shown in Table 1. A total of 28 (80%) samples were from males, and 7 samples were from females. The average age of the patients was 63.5 years old (ranging from 41 to 82 years old). The most common sites of metastasis in this study, in decreasing order of frequency, were pleural cavity, bone, liver, and central nervous system (CNS) (Table 1).

Of the 35 samples, seven (20%) 95% CI: 8.4%-36.9% had EML4-ALK fusion rearrangements. In both EML4-ALK fusion positive and negative groups, the male sex was predominant (Table 2).

There were 6 (85%) male samples in the EML4-ALK rearrangement-positive group and 22 (79%) male samples in the EML4-ALK rearrangement-negative group. In this study, one female patient had positive EML4-ALK rearrangement. The average age of patients with EML4-ALK rearrangement-positive samples was 71 years old, while the average age of patients with a negative sample was 62 years old. One patient in the positive group was younger than 60 (59 years old), while 6 others were 60 years of age or older.

## 4. Discussion

The rearrangement of ALK defines a molecular subset of NSCLC. A previous study in Hong Kong showed the prevalence of the ALK rearrangement in NSCLC to be 4.9%. However, this study involved patients who were not previously tested for the EGFR mutation. In the Hong Kong study, 46.9% of the samples were later found to be EGFR positive [13]. A study by Koivunen et al. showed the same trend with unselected NSCLC samples. In 1% of American and 3% of Korean patients, the EML4-ALK fusion rearrangement gene was identified [14]. This showed that the rearrangement of EML4-ALK is relatively rare (1–5%) in the unselected NSCLC population. However, a study by Shaw et al. in the U.S. showed that in selected adenocarcinoma patients with a light smoking/nonsmoking history, the prevalence of the EML4-ALK rearrangement is as high as 22%. The prevalence is 33% in light smokers/nonsmokers without an EGFR mutation [6]. Study by Ren et al. also showed similar prevalence in which EML4-ALK rearrangement can be as high as 32% among nonsmoking, lung adenocarcinoma without EGFR mutation patients [15]. In addition, Hou et al. also showed 19.8% of EML4-ALK rearrangement in patients with wild-type EGFR [16]. In line with these studies, we showed that the prevalence of EML4-ALK is 20% in our cytology specimens of lung adenocarcinoma with EGFR mutation negative. The EML4-ALK fusion gene is more common in adenocarcinoma than in other lung subtypes. Previous studies showed that EML4-ALK fusion is mutually exclusive with other carcinogenic factors such as EGFR, ROS1, KRAS, and other genes. EML4-ALK is, therefore, almost exclusive to adenocarcinoma [3, 17, 18]. This indicates that due to the exclusivity of EML4-ALK, studies that do not exclude nonadenocarcinoma subtypes and positive EGFR may result in low ALK rearrangement prevalence. The prevalence of EGFR mutation in unselected lung cancer in Indonesia is about 44% [8]. Therefore, the high EML4-ALK prevalence in our study may be explained by our inclusion criteria. This study included only lung adenocarcinoma and did not include EGFR mutation-positive samples.

In this study, the average age of positive and negative ALK rearrangement patients was 71 and 62 years old, respectively. This finding differs from previous studies, which suggested that rearrangements with EML4-ALK were more common in patients around 60 years old. Nevertheless, these studies also showed that the range was quite wide, occurring in patients from 21 to 89 years old [6, 19]. Studies on both Western and Asian populations have shown a similar pattern [6, 13, 14]. Another study showed that age is an independent factor in predicting the frequency of ALK-positive cancer [20]. However, another previous study found that a diagnosis of more advanced disease is more likely in younger patients than in older patients. This suggests that in younger patients, a more progressive biology is seen. Accordingly, early-stage disease patients who were ALK rearrangement positive exhibited an older median age than patients with late-stage disease [20]. The stage of lung cancer is not, however, recorded in our study.

Among the 35 samples, six of the seven positive samples were from male patients. This may be the result of the greater number of male (28 samples) than female samples (7 samples). Samples from females and adenocarcinoma samples are usually tested for EGFR as a first-line test and are more likely to be positive. This explains the high number of male adenocarcinoma samples included in this study. This is also in line with a previous study in which the rearrangement of EML4-ALK was found to be more prevalent in males, despite the study including more female samples [6]. However, a study with a larger sample size suggested no gender difference [3]. The findings of Wang et al. also showed that there is no association between gender and the occurrence of the ALK fusion gene [21]. In line with the previous studies, it can be speculated that there is no gender difference in the incidence of the ALK fusion gene in the Asian population.

A previous study showed that the main sites of NSCLC metastasis include the brain, bone, liver, adrenal glands, thoracic cavity, and distant lymph nodes [22]. A study by Mendoza et al. showed that pleural effusion is the most frequent metastasis in patients with ALK rearrangement, followed by bone, brain, liver, distal lymph node, and other organs [23]. The pattern of this metastasis may be influenced by the fusion variant of EML4-ALK as described by Christopoulos et al. The V3 variant is shown to influence the natural history of the disease, including the metastasis pattern [24]. The metastatic findings in this study were similar to the findings of previous research. However, EML4-ALK fusion variant detection is not performed in this study because of limited resources. This may suggest a similar variant of fusion as shown in these studies, but further testing is required to confirm the presence of this variant.

Several methods exist to detect ALK rearrangement, including qRT-PCR, fluorescence in situ hybridization (FISH), immunohistochemistry (IHC), and next-generation sequencing (NGS). Among these, the positive rate of qRT-PCR is the highest [25]. In our study, the method of detection used was qRT-PCR. In previous studies, FISH was recommended as the method of choice for ALK rearrangement detection [7, 26]. However, FISH detection has limitations including high cost, signal instability, and scoring difficulties. FISH is not easily implemented for widespread use. Therefore, finding a method other than the FISH method to detect the EML4-ALK gene is very important. The IHC method is practical and rapid and can be used to screen for EML4-ALK effectively. However the sensitivity of IHC is shown to be variable. In this study, qRT-PCR is best suited for cytology samples due to unavailability of tissue samples for IHC. The use of IHC for EML4-ALK detection in the availability of tissue samples may still be considered. On the other hand, qRT-PCR is highly sensitive and specific for EML4-ALK detection. Nevertheless, this method requires high-quality samples and can cause false-negative results. The false-negative result may be due to the inability to detect unknown or undetectable ALK fusion gene [3].

PCR-based assays are not as widely used for NSCLC and ALK testing as are FISH and IHC. However, qRT-PCR has significant advantages over FISH and IHC. Normally, qRT-PCR shows the exact variant of the rearrangement and, hence, provides definitive evidence of ALK fusion. FISH and IHC detect relatively indirect signs of the presence of ALK translocation. Furthermore, with high sensitivity and specificity and rapid turnaround time and ease of analysis, qRT-PCR can detect as few as 1% of ALK-driven NSCLC cells [27]. Previous studies have shown that qRT-PCR is an effective method for detecting ALK rearrangement using cytology samples from patients with primary lung cancer [16, 28–30]. The concordance level between different methods ranges from 99 to 100% in these studies. This method of detection in cytology specimens is particularly useful when tissue samples are not available [30]. The apparently better performance of ALK analysis can be explained by the design of the appropriate assay, which will amplify a fusion PCR fragment even in the presence of a significant excess of normal cells [5]. qRT-PCR, compared to other detection methods, can identify only previously well-described fusion partners. However, this approach should be considered to be used in a limited cell number of cytology specimen condition and is better than not testing [11].

In our study, the samples used were direct smear preparations from transthoracic needle aspiration, which differs from most of the previous studies that used surgical specimens [13, 14, 17, 18]. Transthoracic needle aspiration has been shown to be safe and effective in obtaining adequate tissue for genomic profiling [31]. Good concordance is demonstrated between cytology-based and histology-based testing for ALK [5, 10]. Cytology samples such as direct smear preparations provide a high overall tumour fraction and higher average yields of DNA/RNA than samples from cell blocks [10]. Direct smear preparations do not go through formalin, which has been shown to decrease the quality of DNA/RNA [32]. The smears are first rinsed in alcohol and then scraped into eppendorf tubes after typical xylene coverslip removal. DNA/RNA was extracted without destaining beforehand [10]. This suggests that the ALK fusion rearrangement gene can be examined by a less invasive procedure such as transthoracic needle aspiration, which is widely performed in Indonesia.

Notably, to the best of our knowledge, this is the first study on the prevalence of EML4-ALK mutations in Indonesian NSCLC patients. Our study has several limitations that should be acknowledged. Firstly, EML4-ALK rearrangements were detected using qRT-PCR, which identifies only specific EML4-ALK rearrangements. qRT-PCR cannot detect all the variants of ALK fusion genes, including KIF5B-ALK and TFG-ALK. Second, our analysis is based on a relatively limited number of samples. This is because NSCLC adenocarcinoma samples were screened for EGFR beforehand. Even if the EGFR mutation is negative, due to the small number of remaining tumour cells available for analysis, samples often cannot be tested for ALK.

## 5. Conclusions

ALK fusion detection is recommended as category 1 testing in the NCCN guidelines for NSCLC along with EGFR and programmed death-ligand 1 (PD-L1) [7]. To the best of our knowledge, ours is the first study on the prevalence of EML4-ALK mutations in Indonesian NSCLC patients. In addition, this study used cytology specimens, which are commonly used in countries with limited funds available. In these countries, the cost-effectiveness of a biopsy modality is of concern, and a low-cost alternative is often preferred. Cytology techniques are minimally invasive, and cytology samples have been previously demonstrated to be reliable in mutational studies. This study also suggests that PCR using cytology samples might be useful, especially when tissue samples are not available. This study shows that EML4-ALK rearrangement gene has high prevalence in selected adenocarcinoma and EGFR mutation-negative populations. This study is expected to pioneer the development of optimal diagnostic strategies in Indonesia. Therefore, ALK inhibitors in lung cancer can be openly considered for use in Indonesian patients to improve the outcome of this subset of patients. These treatments have been minimally used due to the lack of studies that emphasize the ALK fusion rearrangement gene.

## Figures and Tables

**Table 1:** Characteristics of the study samples.

Characteristics	*N*
Gender	
Male (%)	28 (80)
Female (%)	7 (20)
Age, y	
Average (range)	63.5 (41–82)
Metastasis site	
Pleura	16
Bone	7
Liver	4
CNS	1

**Table 2:** EML4-ALK rearrangement status.

Characteristics	EML4-ALK rearrangement (+)	EML4-ALK rearrangement (-)
	*N*	*N*
EML4-ALK status (%)	7 (20)	28 (80)
Sex		
Male (%)	6 (85)	22 (79)
Female (%)	1 (15)	6 (21)
Age		
Average	71	62
<60 years old	1	11
≥60 years old	6	17

## Data Availability

The data used to support the findings of this study are available from the corresponding author upon request.
